# Hospitalization determinants in patients with Mpox disease: The CEME-22 Project

**DOI:** 10.1016/j.heliyon.2024.e30564

**Published:** 2024-05-01

**Authors:** G. Ramírez Olivencia, M.M. Vera García, M. Velasco Arribas, J. Casabona, M.J. Martínez, F.J. Membrillo De Novales, on behalf of the CEME-22 Study Group. Study Group team members are listed in the Acknowledgments

**Affiliations:** a Hospital Militar Central Gómez Ulla, Spain; b Centro Sanitario Sandoval, Spain; c Hospital Universitario Fundación Alcorcón, Spain; d Centro de Investigación Biomédica en Red de Epidemiología y Salud Pública (CIBERESP), Instituto de Salud Carlos III, Spain; e Hospital Clínic de Barcelona, Spain

**Keywords:** Mpox disease, Hospitalization, Immunocompromised, Healthcare facilities, Outbreak

## Abstract

**Objectives:**

This sub-analysis seeks to delineate and characterize factors influencing hospitalization in individuals diagnosed with Mpox disease amidst the initial outbreak in Spain in the onset of 2022.

**Methods:**

Employing a non-probabilistic convenience sampling approach, a retrospective multicenter investigation was carried out to examine Monkeypox virus infection within Spanish healthcare facilities.

**Results:**

The median duration of the disease was 16 days, with 4.2 % of cases resulting in hospitalization. There was a single ICU admission leading to fatality. Sequelae were observed in 2.3 % of cases. Multivariate analysis revealed that hospitalization decisions were influenced by immunosuppression and severe symptoms, including gastrointestinal, neurological, ear-nose-throat, and respiratory manifestations. Significant analytical parameter differences were restricted to hemoglobin levels at diagnosis.

**Conclusions:**

This study elucidates factors influencing hospitalization decisions for Monkeypox patients in Spain, emphasizing the importance of immunosuppression and extracutaneous symptoms involving the gastrointestinal, ear-nose-throat, and respiratory pathways. In summary, hospitalization determinations arise from the interplay of these crucial dimensions.

## Highlights

• Sequelae of Mpox disease were observed in 2.3 % of cases and one death was reported.
• Less than 5 % of patients were hospitalized during early Spanish Mpox epidemic.
• Immunosuppression and severe symptoms determined hospitalization.

## 1 Introduction

Since the 1970s, the Mpox virus (MPXV) disease has been recognized for its impact on humans in Central Africa, characterized by the emergence of a distinctive rash. This infectious ailment unfolds in a sequence that encompasses a prodromal phase typified by fever, weakness, lymph node swelling, and intermittent headaches. Subsequently, the disease advances to present a centrifugal rash that intriguingly mirrors the pattern observed in smallpox infections. This characteristic rash metamorphoses through a spectrum of stages, including macules, papules, vesicles, pseudopustules, crusts, and scabs, ultimately subsiding within a timeframe of 7–14 days [1]. Crucially, disease severity shows a direct correlation with lesion count, with noticeable differences between Clade I (Central African) and the milder Clade II (West African) [2,3].

However, in a departure from its historical geographic confines, Mpox has undergone a clear re-emergency, affecting regions both endemic and non-endemic. A notable turning point appeared in May 2022 when an unforeseen outbreak swept across Western continents, stretching its impact from Europe to the Americas and encompassing all six WHO regions. Of particular significance, cases arose in regions far removed from traditional epicenters, thereby underscoring significant shifts in transmission patterns and instigating profound global public health concerns [[4], [5], [6], [7]]. This sudden expansion resulted in an estimated 87 thousand reported cases, accompanied by the heart-wrenching loss of 112 lives across 110 countries. Furthermore, the repercussions of the outbreak were exacerbated by its unequal effect on specific demographics, particularly among homosexual, bisexual, and other individuals engaging in same-sex relations, underscoring the intensified transmission patterns within interconnected sexual cohorts. Given this expansive geographical reach and epidemiological characteristics, a comprehensive investigation is imperative to decode the intricacies of this rapidly evolving scenario.

Moreover, the resurgence of Mpox as a worldwide health concern prompts inquiries regarding potential alterations in viral characteristics and alterations in human conduct, impacting transmission routes, reservoirs, and control methodologies. Efficient resource allocation during emergent or re-emergent infectious diseases, exemplified by the recent COVID-19 pandemic, is paramount. Studies exploring ambulatory strategies for severe COVID-19 underscore the urgent need to establish clear criteria for hospitalization versus ambulatory care [8,9]. This imperative extends beyond COVID-19 to other infectious diseases, necessitating robust research, including prospective trials and cost-effectiveness analyses, to guide evidence-based decision-making. Comprehensive clinical guidelines and decision support tools are essential for effective patient triaging by healthcare providers.

Optimizing care pathways ensures judicious resource utilization and enhances patient outcomes. Considering the disease's wide-ranging impact on diverse regions and demographics, understanding the intricacies that shape the decision to hospitalize individuals afflicted by Mpox becomes a pivotal and intricate area of investigation. Consequently, against the backdrop of the emerging Mpox outbreak that unfolded in May 2022, the SEIMC (The Spanish Society for Clinical Microbiology and Infectious Diseases) embarked on the nationwide CEME-22 study [10], thereby creating the framework for the present sub-study to unravel these pivotal determinants.

This sub-study aims to identify and describe determinants of hospitalization among patients affected by Mpox disease during the early 2022 outbreak in Spain.

## 2 Methods

The SEIMC carried out an observational, retrospective, multicentre study aimed at comprehensively characterizing cases of Mpox virus infection among patients in various healthcare settings across Spain. This research employed a non-probability convenience sampling method, actively involving its members. In this method, individuals are included in the sample based on their ready availability and accessibility, often due to their proximity or ease of recruitment, with patients being consecutively enrolled. The study population included individuals who received medical care at the enlisted institutions, comprising primary care facilities, general hospitals, and STI clinics. Researchers curated distinct instances from each registered facility. Data encompassing demographics, epidemiology, clinical presentations, and biological markers were extracted from medical records and linked information systems by means of a unique personal identifier (UPI) and a specific form. This approach facilitates a thorough examination of interrelationships and dependencies. In the management of individuals with emergent or atypical illnesses, hospitalization for treatment or observation to monitor for complications is commonly considered. This distinction can significantly influence patient management compared to outpatient care, particularly relevant given recent experiences with COVID-19.

### 2.1 Inclusion criteria

Enrolled participants in this study had clinical specimens analyzed before July 13th, 2022, revealing a positive MPXV genome result via PCR or sequencing. Validation as an Mpox case required meeting the three conditions per the Spanish Ministry of Health guidelines (latest revision as of July 2022). Notably, the initial selection prioritized patients with a positive PCR, subsequently evaluated against all three criteria.

### 2.2 Clinical criterion

A vesicular rash on any part of the body, along with one or more characteristic signs or symptoms of Mpox infection (such as acute febrile illness - exceeding 38.5°C-, muscle discomfort, arthralgia, lumbar pain, adenopathy severe or headache), subsequent to the exclusion of alternative pathologies.

### 2.3 Epidemiological criterion

If an individual fulfils any of the following criteria within 3 weeks before the onset of symptoms.

• Close contact with a confirmed or actively investigated Mpox case.
• Involvement in activities associated with heightened sexual risk.
• Travel history to West or Central Africa, recognized as endemic areas with confirmed viral circulation.

### 2.4 Laboratory standard

Identification of the MPXV genome in a clinical specimen via PCR or sequencing.

### 2.5 Exclusion criteria

Excluded from the study were individuals who met the eligibility criteria but were below 18 years of age.

### 2.6 Definitions

1. Immunologically Compromised Patients: This classification pertains to individuals diagnosed with primary immunodeficiency, HIV infection, those prescribed immunosuppressive medication, or those affected by any medical condition causing either temporary or persistent impairment of the immune system, thereby increasing vulnerability to the particular disease under consideration.
2. Pre-existing Smallpox Vaccination: This criterion is met when either a) an official vaccination certificate can be provided as evidence; or b) an identifiable scar consistent with vaccination is observed.
3. Healing. During the period of study, definitive criteria for disease remission remained absent, with only patient isolation protocols delineated. In our study, patient recovery was defined by the confluence of the following conditions:
a) Resolution of skin eruption, signaled by the shedding of crusts from all lesions. Once all crusts have sloughed off, and new skin has formed, healing is deemed achieved. Closure of fistulizing lymph node ulcers is imperative for infection resolution. Potential scarring with dimples or variations in skin pigmentation might persist.
b) Entire cure of mucosal manifestations, including conjunctivitis, pharyngitis, and oral and proctitis ulcers.
  c) Full recovery of general symptoms (systemic recovery), encompassing fever, perspiration, and chills.
d) Absence of progression in lymphadenopathy or neurological manifestations.
e) Extracutaneous or extramucosal manifestations attributed solely to secondary complications, with alternative etiologies substantiated (e.g., respiratory superinfection in intubated patients; renal insufficiency due to cidofovir, etc.). Even if stemming from diverse etiologies (e.g., bacterial abscesses), cutaneous or mucosal manifestations resultant from secondary complications will be classified as active disease.
4. Conversely, failure to meet these criteria delineated the presence of residual sequelae.

Diverse analytical methodologies were employed to scrutinize the dataset, with each technique tailored to the unique attributes of the data under examination. For datasets demonstrating a Gaussian distribution, characterization of quantitative data was achieved by computing both the mean and the standard deviation. Conversely, when confronted with datasets manifesting non-Gaussian or skewed distributions, the assessment centered on the median and percentiles. Qualitative data, on the other hand, underwent a process of synthesis, involving the presentation of absolute frequencies alongside their corresponding relative proportions. This approach ensured a comprehensive exploration of the data landscape. In the univariate analysis, hospital admission (or sequelae) served as the dependent variable, while age, sex, transgender status, risk factors, prior smallpox vaccination, immunosuppression, HIV status, skin lesions, digestive, neurological, ENT, and respiratory symptoms acted as independent variables. In the multivariate analysis, hospital admission (or sequelae) served as the dependent variable, while age, risk of exposure (sexual vs others), prior smallpox vaccination, immunosuppression, and severe symptoms acted as independent variables.

### 2.7 Variables

The investigation encompassed the compilation of demographical and epidemiological data, with a specific focus on factors such as transmission mechanisms, historical records of smallpox vaccination, and travel history. A thorough compilation of clinical particulars was systematically recorded, offering insights into immunodeficiency conditions and covering manifestations across dermatological, pulmonological, gastrointestinal, urological, neurological, ENT and systemic presentations. The dataset was additionally augmented by the inclusion of pertinent hospitalization data. The variables underwent meticulous delineation, with the dependent variable designated as "Hospitalization," serving as the central point of analysis. Concurrently, a substantial array of "Epidemiological and Clinical-Analytical Data" was identified as the independent variables. This methodical classification not only yielded a comprehensive perspective but also established a structured framework for investigating the intricate relationship between instances of hospitalization and a diverse spectrum of pertinent epidemiological and clinical factors.

#### 2.7.1 Ethical-legal aspects

This retrospective observational study conformed to the requirements delineated in Royal Spanish Decree 957/2020 concerning pharmaceuticals. The preservation of personal data confidentiality was ensured through anonymization. The database underwent dissociation, and the principal registry maintained no identifiable patient information. The patient data was entered into an electronic data capture tool integrated within a certified network database acknowledged as RedCap. In accordance with Article 13.3 of the European General Data Protection Regulation, Regulation (EU) 2016/679, due consideration was given. The study garnered approval from the pertinent Ethics Committee (FSG 023-22. Comité ético de investigación con medicamentos CEIm del Hospital Universitario Fundación Alcorcón de Madrid), with an exemption granted for informed consent; non-inclusion of this aspect would have introduced substantial bias to the study. Upon obtaining approval, concurrence was sought from the other contributing centers. The researchers were firmly committed to upholding human rights and dignity in biological and medical research, guided by ethical standards. This encompassed adherence to the Good Clinical Practice Guidelines, the Helsinki Declaration and the Oviedo Convention as stipulated by the Spanish Agency for Medicines and Medical Devices (AEMPS).

## 3 Results

### 3.1 Socio-demographic and epidemiological characteristics of participants at baseline

Factual data from the National Network of Epidemiological Surveillance (RENAVE) by July 22, 2022, documented 3125 confirmed MPX cases nationwide. Concurrently, a cohort of 1472 individuals was enlisted for the study, exhibiting an average age of 38.6 years (standard deviation: 9.3). The demographic attributes of the cohort, encompassing gender, birthplace, residency, and potential exposure, are delineated in Table 1.

**Table 1:** Characteristics of the included population in the CEME-22 study.

		n (%^a^)
Sex	Male	1450 (99)
	Female	15 (1)
Gender	Cisgender	1459 (99.7)
	Transgender	4 (0.3)
Country of birth	European Region	880 (64.14)
	Region of Americas	466 (33.97)
	African Region	18 (1.31)
	Eastern Mediterranean Region	3 (0.22)
	South-East Asia Region	1 (0.07)
	Western Pacific Region	4 (0.29)
Country of residence	European Region	1449 (99.38)
	Region of Americas	8 (0.55)
	African Region	1 (0.07)
Suspected exposure	Sexual	1385 (96.4)
	Occupational^b^	6 (0.4)
	Other^c^	45 (3.1)
Immunosuppression	HIV^d^	549 (98.6)
	Drug-induced	3 (0.5)
	Hematological disease	1 (0.2)
	Unclassified	4 (0.7)

a Percentages are based on cases with available data.

b Occupational category comprises healthcare workers, tattoo parlor, and sauna staff, etc. No reported cases among healthcare personnel.

c Other suspected exposure includes: cohabitation, non-sexual close contact, participation in contact sports ….

d Median CD4 lymphocyte counts: 724 cells/μL (IQR: 541–984; n = 344). Viral load data was largely unavailable.

### 3.2 Clinical presentation

When data were accessible, 1382 subjects (95.7 %) exhibited an exanthem, with the rash evident prior to diagnosis in 999 instances (84.5 %). Patients without a pre-diagnosis rash underwent evaluation for other relevant symptoms (general, gastrointestinal, respiratory, or ear, nose, and throat -ENT-), while also meeting epidemiological criteria. Fever was documented in 691 cases (48.2 %), followed by lymphadenopathy in 634 (44.4 %), myalgias in 295 (20.7 %), rigors/chills in 156 (11.0 %), arthralgia/arthritis in 128 (9.0 %), diaphoresis in 49 (3.5 %), and dorsalgia in 19 (1.3 %). These symptoms preceded diagnosis in the majority of cases, ranging from 68.5 % to 91.8 %.

The most prevalent gastrointestinal symptoms observed were proctitis (241 instances, comprising 17 %) and odynophagia (240 instances, representing 16.9 %). These were followed by diarrhea (75 cases, accounting for 5.3 %), abdominal discomfort (37 cases, 2.6 %), dysphagia (34 cases, 2.4 %), and nausea and/or vomiting (28 cases, 2.0 %).

The predominant neurological manifestation observed was headache, present in 210 instances (14.7 %), with a preceding occurrence noted in 86.7 % of these cases. Singular occurrences were noted for neck stiffness, while neither seizures nor confusion syndrome were reported. Additionally, ENT symptoms were documented, including oral ulcers in 95 cases (6.7 %), nasal congestion in 23 cases (1.6 %), and otalgia in 7 cases (0.5 %).

Regarding respiratory symptoms, dyspnea was noted in 11 instances (0.8 %), cough in 39 (2.7 %), and chest pain in 6 (0.4 %). Additionally, conjunctivitis manifested in 11 occurrences (0.8 %), urethritis in 79 (5.6 %), and paraphimosis in 20 (1.4 %). All percentages are based on available data.

### 3.3 Diagnostic findings

Out of 1472 cases, 1387 (94.2 %) were confirmed as MPX by PCR analysis of cutaneous lesions. Negative results were observed in 5 cases (0.4 % of total cases), while the test was not conducted on cutaneous specimens in 80 instances (5.4 %). Additional fluid specimens, including urine, blood/serum, or pharyngeal, rectal, and urethral swabs, underwent PCR analysis for the remaining diagnoses.

### 3.4 Evolution of the disease and hospitalization

Data on disease progression were unavailable for 163 patients. Healing was observed in 1269 cases, accounting for 96.9 % of the cases with IA. The disease had a median duration of 16 days, with an interquartile range spanning 9–23 days.

Hospitalization details were accessible for 1378 patients, resulting in 58 admissions, which represented 4.2 % of the IA cases. Remarkably, among these admissions, only one case required intervention in the intensive care unit (ICU); this patient finally died.

Post-effects information was unavailable in 196 cases, while sequelae were documented in 29 cases (including 3 hospitalized cases, one of which resulted in death), comprising 2.3 % of the IA cases. Among the spectrum of aftermath, the most frequent occurrences were scars (11/29), hyperpigmentation (5/29), and proctitis/anal pain (5/29). Additional distinctive sequelae included persistent ulcers, phimosis, impetigo, cellulitis, keratitis, and local reduced sensitivity.

The univariate analysis examined two dependent variables: hospitalization, and presence of sequelae. This analysis considered a spectrum of independent variables, encompassing epidemiological and clinical factors (Table 2), as well as analytical parameters (Table 3).

**Table 2:** Note: The outlined concepts encompass a spectrum of manifestations. Skin symptoms include rash and/or abscess. Digestive symptoms cover diarrhea, dysphagia, abdominal pain, nausea, vomiting, odynophagia, and/or proctitis. Neurological symptoms encompass headache, seizures, stiff neck, and/or altered mental status. ENT symptoms (Ear, Nose, and Throat) entail congestion, otalgia, and/or oral ulcers. Respiratory symptoms involve dyspnea, wheezing, cough, and/or chest pain. Additional symptoms encompass conjunctivitis, paraphimosis, and/or urethritis. Severity was defined as instances where hospitalization, including ICU admission, occurred, or when sequelae or mortality were observed. Information available in 1378 (*) and 1276 (**) respectively. NA: Not applicable. p-values from univariate analysis.

			Hospital admission*		p-value	Secuelae**		p-value
			No	Yes		No	Yes	
			1320 (90.5 %)	58 (9.5 %)		1247 (94.5 %)	29 (5.5 %)	
**Age (years)**	**Mean**		38.7	39.5	.671	38.7	41.0	.298
	**Std. Deviation**		9.4	9.8		9.4	10.6	
**Sex**	**Man**	n	1307	58	1.000	1235	28	.259
		%	95.8	4.2		97.8	2.2	
	**Woman**	n	13			12	1	
		%	100.0			92.3	7.7	
**Transgenderism**		n	4		1.000	4		1.000
		%	100.0			100.0		
**Risk exposure**	**Occupational/other**	n	48	2	1.000	47	1	1.000
		%	96.0	4.0		97.9	2.1	
	**Sexual**	n	1245	55		1171	28	
		%	95.8	4.2		97.7	2.3	
**Prior smallpox vaccination**		n	69	4	.553	68	2	.652
		%	94.5	5.5		97.1	2.9	
**Immunocompromised**		n	496	34	.**001**	496	11	.845
		%	93.6	6.4		97.8	2.2	
**HIV infection**		n	700	19	.**003**	630	14	.811
		%	97.4	2.6		97.8	2.2	
**Skin symptoms**		n	1264	56	1.000	1193	29	.629
		%	95.8	4.2		97.6	2.4	
**Digestive symptoms**		n	446	39	.**000**	432	12	.503
		%	92.0	8.0		97.3	2.7	
**Neurological symptoms**		n	187	16	.**005**	173	7	.173
		%	92.1	7.9		96.1	3.9	
**ENT symptoms**		n	102	16	.**000**	99	6	.**021**
		%	86.4	13.6		94.3	5.7	
**Respiratory symptoms**		n	36	10	.**000**	43	2	.255
		%	78.3	21.7		95.6	4.4	
**Others**		n	86	9	.**017**	86	5	.**048**
		%	90.5	9.5		94.5	5.5

**Table 3:** Biochemical and hematological parameters at diagnosis. Information available in 1378 (*), and 1276 (**) respectively.

*At diagnosis*	Hospital admission*			Secuelae**		
	No	Yes	p-value	No	Yes	p-value
**Levels of hemoglobin <12 g/dl; n (%)**	10 (2.6)	6 (11.3)	**0.008**	13 (3.3)	1 (6.7)	**0.040**
**Total leukocytes > 11000 cell/mm3; n(%)**	46 (12)	9 (17)	0.303	48 (12.2)	2 (13.3)	0.705
**Total neutrophils > 7500 cell/mm3; n(%)**	26 (76.5)	8 (23.5)	0.053	29 (7.7)	1 (7.1)	1.000
**Total lymphocytes < 1000 cell/mm3; n(%)**	34 (9.2)	4 (7.7)	1.000	33 (8.7)	2 (14.3)	0.361
**Total platelets < 130000 cell/mcl; n(%)**	12 (3.2)	2 (3.8)	0.680	13 (3.4)		1.000
**Levels of AST liver enzyme >34 UI/l; n(%)**	85 (31)	13 (36.1)	0.537	86 (31)	6 (54.5)	0.111
**Levels of ALT liver enzyme >45 UI/l; n(%)**	69 (21)	11 (27.5)	0.349	74 (22.2)	3 (30)	0.699
**Total bilirubin >1.2 mg/dl; n(%)**	20 (7.2)	2 (5.3)	0.756	20 [7]	1 (12.5)	0.450
**Levels of creatinine > 1.3 mg/dl; n(%)**	11 (2.8)	5 (9.6)	**0.029**	15 (3.8)	1 (7.1)	0.432
**Levels of urea > 49 mg/dl; n(%)**	10 (3.7)	2 (4.7)	0.674	12 (4.3)		1.000

In the univariate analysis, factors influencing the decision for hospitalization were identified. These factors included immunosuppression due to any cause and the presence of extracutaneous symptoms such as gastrointestinal involvement, neurological symptoms, ear-nose-throat symptoms, respiratory symptoms, or other types of complications (conjunctivitis/paraphimosis/urethritis). In the analysis of analytical parameters, statistically significant differences were found solely in relation to hemoglobin levels <12 g/l and creatinine >1.3 mg/dl at the time of diagnosis.

The results of the multivariate analysis are presented in Table 4. The multivariate analysis found no association between any epidemiological or clinical variables and the development of sequelae.

**Table 4:** Note: Severe symptoms include cutaneous abscess, proctitis, seizures, stiff neck, altered mental status, dyspnea, wheezing, and/or chest pain. Information available in 1378 (*), 1276 (**) and 1381 cases (***) respectively. NA: Not applicable.

Hospital Admission*	Multivariate Analysis		
	OR	IC95 %	p-value
**Age (for 1 year)**	1	0.76–1.04	0,802
**Risk exposures (sexual vs others)**	0.48	0.11–2.19	0.344
**Prior smallpox vaccination**	0.93	0.25–3.47	0.917
**Immunocompromised**	**2.46**	**1.27**–**4.76**	**0.007**
**Severe symptoms**	**6.08**	**3.12**–**11.87**	**< 0.001**
Secuelae**	Multivariate Analysis		
	OR	IC95 %	p-value
**Age (for 1 year)**	1.04	0.98–1.09	0.193
**Risk exposures (sexual vs others)**	N.A.	N.A.	N.A.
**Prior smallpox vaccination**	1.34	0.27–6.78	0.721
**Immunocompromised**	0.806	0.29–2.23	0.678
**Severe symptoms**	0.709	0.20–2.50	0.592

## 4 **Discussion**

Our study's robustness stems from its extensive sample size, encompassing a substantial cohort of patients from the initial phase of the pandemic in Spain (CEME-22). This enables a dependable portrayal of the healthcare interventions administered during that period.

The decision to hospitalize a patient depends on three main factors: the illness type, the individual's health, and the healthcare context. In our study, admission was primarily based on immunosuppression and symptom severity. Notably, CKD, prior pulmonary disease, and other comorbidities were not initially included in our data collection process. Nevertheless, these factors could potentially impact the results. Given the demographic composition of the sample in terms of sex and age, it is conceivable that the impact of these variables on hospitalization decisions and the onset of sequelae may not have been fully elucidated. The identical circumstances could account for the absence of disparities in exposure pathways and prior vaccination. It is imperative to consider that, during that period of the epidemic, variola vaccination had not been implemented.

Previous mortality data from pre-2022 outbreaks hinted at potential complications and high fatality rates during pandemics [3]. However, our findings unequivocally show lower mortality and reduced hospitalization rates [[11], [12], [13]]. Local treatments availability, which helps mitigate cutaneous complications [[14], [15], [16]], alongside limited access to medications like tecovirimat [17,18] have significantly decreased the need for hospitalization, enabling outpatient management. Statistical differences observed regarding anemia and renal insufficiency weren't clinically relevant. In Spain, hospital admission wasn't always necessary for isolation; home isolation was encouraged.

In infectious diseases, hospital admission is often determined by concurrent illnesses or comorbidities rather than the severity of the infection itself. The population in our study is relatively young and healthy, with STIs, mainly HIV, as the primary associated illnesses. Within this context, immunosuppression emerges as a primary determinant, particularly in cases linked to HIV infection [[19], [20], [21]]. The presence of immunosuppression, acting as a modulating factor in disease progression, underscores its pivotal role in shaping hospitalization decisions. While some studies, such as Shin's systematic review [22], highlight distinct clinical features associated with mucosal contact in individuals living with HIV, others present contrasting findings [23]. In our study, HIV infection, even alone and often irrespective of CD4 counts, significantly influenced the decision for hospital admission.

Complications also favor hospital admission, particularly those impairing basic functions like swallowing or breathing. Although some complications were rare in our study, they have been previously linked to unfavorable outcomes [[24], [25], [26], [27]]. However, non-vital complications like ocular involvement [[24], [25], [26], [27]] or proctitis [28,29] likely lead to admission for poorly controlled symptoms or close monitoring. The predominantly cutaneous presentation of Mpox in this outbreak may explain the absence of identifiable analytical markers linked to hospitalization or sequelae.

In some cases, a patient's need for hospitalization depends on the treatment facility's capabilities. For example, primary care centers may lack resources for complex illnesses, necessitating transfer to specialized hospitals. However, Spain's healthcare system ensures seamless patient transfers, guaranteeing access to necessary medical care regardless of initial treatment location. This interconnected system, facilitated by medical professionals' coordination, ensures personalized and timely treatment for each patient.

The primary limitations of the study encompass its retrospective design, incomplete availability of analytical data across cases, and partial representation of the disease outbreak wave.

In summary, limited knowledge during the first wave of Mpox in Spain led clinicians to prioritize hospitalization for patients with immunosuppression or severe symptoms. However, all Mpox cases require individualized assessment, considering all factors within the specific outbreak context. Our study illuminates the recovery outcomes of Mpox patients, emphasizing a significant proportion achieving favorable prognoses, even among those hospitalized (98.3 %). Particularly noteworthy is the minimal incidence of sequelae (3.4 %), accentuating the viability of ambulatory treatment strategies. This aligns seamlessly with the imperative to refine care settings, ultimately enhancing patient outcomes. The availability of local treatments and access to oral medications have substantially reduced hospitalization rates for Mpox disease, enabling outpatient management and easing the strain on healthcare facilities. Based on the conclusions of our research, future studies should evaluate different approaches for local complications such as ocular involvement or ambulatory management of proctitis. Moreover it is mandatory explore other risk factors that may influence the need for hospitalization in Mpox patients, in addition to immunosuppression and symptom severity.

## 5 Footnotes

- This study was founded by Foundation SEIMC-GESIDA, Spain.
- The authors declare no conflicts of interest.
- This study results were partially presented at ESCMID 2023 and SEIMC 2023 congresses.

## Data availability statement

The data pertinent to our study has not been deposited into a publicly accessible repository. In line with ethical and legal considerations, technological constraints, and respect for intellectual property rights, we have opted against public deposition of our study's data. This decision is aimed at ensuring the proper handling and protection of sensitive information, while also recognizing the limitations of our current technological capacities.

## CRediT authorship contribution statement

**G. Ramírez Olivencia:** Writing – review & editing, Writing – original draft, Visualization, Validation, Supervision, Methodology, Investigation, Formal analysis, Conceptualization. **M.D.M. Vera García:** Writing – review & editing, Methodology, Investigation, Conceptualization. **M. Velasco Arribas:** Writing – review & editing, Methodology, Investigation, Formal analysis, Conceptualization. **J. Casabona:** Writing – review & editing, Methodology, Investigation, Formal analysis, Conceptualization. **M.J. Martínez:** Writing – review & editing, Conceptualization. **F.J. Membrillo De Novales:** Writing – review & editing, Methodology, Formal analysis, Conceptualization.

## Declaration of competing interest

The authors declare the following financial interests/personal relationships which may be considered as potential competing interests: German Ramirez-Olivencia reports administrative support and article publishing charges were provided by Sociedad Española de Enfermedades Infecciosas y Microbiología Clínica (10.13039/501100015756SEIMC). If there are other authors, they declare that they have no known competing financial interests or personal relationships that could have appeared to influence the work reported in this paper.

## References

[1] Petersen E., Kantele A., Koopmans M., Asogun D., Yinka-Ogunleye A., Ihekweazu C. (2019). Human monkeypox: Epidemiologic and clinical characteristics, diagnosis, and Prevention. Infect Dis Clin North Am..

[2] Jezek Z., Szczeniowski M., Paluku K.M., Mutombo M. (1987). Human monkeypox: clinical features of 282 patients. THE JOURNAL OF INFECTIOUS DISEASES.

[3] Bunge E.M., Hoet B., Chen L., Lienert F., Weidenthaler H., Baer L.R. (2022). The changing epidemiology of human monkeypox—a potential threat? A systematic review. PLoS Negl Trop Dis [Internet].

[4] Ee S., Yong F., Ng O.T., Jie Z., Ho M., Mak T.M. (2020). Imported monkeypox, Singapore.

[5] Erez N., Achdout H., Milrot E., Schwartz Y., Wiener-Well Y., Paran N. (2019). Diagnosis of imported monkeypox, Israel, 2018. Emerg. Infect. Dis..

[6] Vaughan A., Aarons E., Astbury J., Balasegaram S., Beadsworth M., Beck C.R. (2018). Two cases of monkeypox imported to the United Kingdom, september 2018. Euro Surveill..

[7] Huhn G.D., Bauer A.M., Yorita K., Graham M.B., Sejvar J., Likos A. (2005). Clinical characteristics of human monkeypox, and risk factors for severe disease. Clin. Infect. Dis..

[8] Wynants L., Van Calster B., Collins G.S., Riley R.D., Heinze G., Schuit E. (2020). Prediction models for diagnosis and prognosis of covid-19: systematic review and critical appraisal. The BMJ.

[9] Fried S., Bar-Shai A., Frydman S., Freund O. (2023). Internal and Emergency Medicine.

[10] Ramírez-Olivencia G., Velasco Arribas M., Vera García M.M., Casabona J., Martínez M.J., Membrillo De Novales F.J. (2024). Clinical and epidemiological characteristics of the 2022 mpox outbreak in Spain (CEME-22 study). Open Forum Infect Dis [Internet].

[11] DeWitt M.E., Polk C., Williamson J., Shetty A.K., Passaretti C.L., McNeil C.J. (2022). Global monkeypox case hospitalisation rates: a rapid systematic review and meta-analysis. EClinicalMedicine.

[12] Li P., Li J., Ayada I., Avan A., Zheng Q., Peppelenbosch M.P. (2023). Clinical features, antiviral treatment, and patient outcomes: a systematic review and Comparative analysis of the previous and the 2022 mpox outbreaks. J. Infect. Dis.228.

[13] Sah R., Mohanty A., Abdelaal A., Reda A., Rodriguez-Morales A.J., Henao-Martinez A.F. (2022).

[14] Sobral-Costas T.G., Escudero-Tornero R., Servera-Negre G., Bernardino J.I., Gutiérrez Arroyo A., Díaz-Menéndez M. (2023). Human monkeypox outbreak: epidemiological data and therapeutic potential of topical cidofovir in a prospective cohort study. J. Am. Acad. Dermatol..

[15] Gamo Guerrero M., Simón Gozalbo A., Martín Díaz M., Díez Madueño K., Del Río Pena E., De la Cueva P. (2023). Interdisciplinary management of mpox-related local complications: report on a series of cases. Front. Med..

[16] Tarín-Vicente E.J., Alemany A., Agud-Dios M., Ubals M., Suñer C., Antón A. (2022). Clinical presentation and virological assessment of confirmed human monkeypox virus cases in Spain: a prospective observational cohort study. Lancet.

[17] Grosenbach D.W., Honeychurch K., Rose E.A., Chinsangaram J., Frimm A., Maiti B. (2018). Oral tecovirimat for the treatment of smallpox. N. Engl. J. Med..

[18] Merchlinsky M., Albright A., Olson V., Schiltz H., Merkeley T., Hughes C. (2019). The development and approval of tecoviromat (TPOXX®), the first antiviral against smallpox. Antivir. Res..

[19] Ortiz-Saavedra B., Montes-Madariaga E.S., Cabanillas-Ramirez C., Alva N., Ricardo-Martínez A., León-Figueroa D.A. (2023).

[20] Mitjà O., Alemany A., Marks M., Lezama Mora J.I., Rodríguez-Aldama J.C., Torres Silva M.S. (2023). Mpox in people with advanced HIV infection: a global case series. Lancet.

[21] Philpott D.C., Bonacci R.A., Weidle P.J., Curran K.G., Brooks J.T., Khalil G. (2023). Low CD4 count or being out of care Increases the risk for mpox hospitalization among people with HIV and mpox. Clinical Infectious Diseases [Internet].

[22] Shin H., Shin H., Rahmati M., Koyanagi A., Jacob L., Smith L. (2023). Comparison of clinical manifestations in mpox patients living with HIV versus without HIV: a systematic review and meta-analysis. J. Med. Virol..

[23] Pilkington V., Quinn K., Campbell L., Payne L., Brady M., Post F.A. (2023). Clinical presentation of monkeypox in people with and without HIV in the United Kingdom during the 2022 global outbreak. AIDS Res Hum Retroviruses.

[24] Billioux B.J., Mbaya O.T., Sejvar J., Nath A. (2022). Neurologic complications of smallpox and monkeypox: a review. JAMA Neurol..

[25] Badenoch J.B., Conti I., Rengasamy E.R., Watson C.J., Butler M., Hussain Z. (2022). Neurological and psychiatric presentations associated with human monkeypox virus infection: a systematic review and meta-analysis. EClinicalMedicine.

[26] Müller-Jensen L., Kriedemann H., Anvari K., Huehnchen P., Siffrin V. (2023). Complicated monkeypox infection in a patient with Multiple Sclerosis and Fingolimod treatment. Neurology.

[27] Pastula D.M., Copeland M.J., Hannan M.C., Rapaka S., Kitani Takashi, Kleiner E. (2022). Morbidity and mortality weekly report two cases of monkeypox-associated Encephalomyelitis-Colorado and the District of Columbia. https://www.cdc.gov/mmwr.

[28] Gandhi A.P., Gupta P.C., Padhi B.K., Sandeep M., Suvvari T.K., Shamim M.A. (2023).

[29] Abdelaal A., Reda A., Hassan A.R., Mashaal A., Abu Serhan H., Katamesh B.E. (2023). Monkeypox-associated manifestations and complications involving the eye: a systematic review and meta-analysis of previous and current outbreaks. Asia-Pacific Journal of Ophthalmology.

